# Pitfalls in the Immunochemical Determination of β-Lactam Antibiotics in Water

**DOI:** 10.3390/antibiotics10030298

**Published:** 2021-03-12

**Authors:** Alexander Ecke, Rudolf J. Schneider

**Affiliations:** 1 BAM Federal Institute for Materials Research and Testing, 12205 Berlin, Germany; alexander.ecke@bam.de; 2 Department of Chemistry, Humboldt-Universität zu Berlin, 12489 Berlin, Germany; 3 Faculty III Process Sciences, Technische Universität Berlin, 10623 Berlin, Germany

**Keywords:** β-lactams, penicillins, hydrolysis, ELISA, immunoassay

## Abstract

Contamination of waters with pharmaceuticals is an alarming problem as it may support the evolution of antimicrobial resistance. Therefore, fast and cost-effective analytical methods for potential on-site analysis are desired in order to control the water quality and assure the safety of its use as a source of drinking water. Antibody-based methods, such as the enzyme-linked immunosorbent assay (ELISA), can be helpful in this regard but can also have certain pitfalls in store, depending on the analyte. As shown here for the class of β-lactam antibiotics, hydrolysis of the β-lactam ring is a key factor in the immunochemical analysis as it influences antibody recognition. With the antibody used in this study, the limit of detection (LOD) in the immunoassay could be significantly reduced by hydrolysis for the five tested penicillins, with the lowest LOD for carbenicillin (0.2 nmol/L) and the greatest impact on penicillins G and V (reduction by 85%). In addition to enhanced quantification, our strategy also provides access to information about the degree of hydrolysis in water samples as shown for the most abundant penicillin amoxicillin.

## 1. Introduction

**Figure 1:**
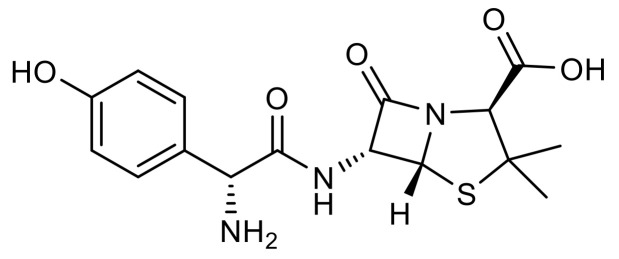
Chemical structure of amoxicillin (AMX).

The overuse of antibiotics in human and veterinary medicine contributes to an increasing discharge of pharmaceuticals and their metabolites into the environment via excretion and disposal [1,2,3,4]. The major concern about antibiotics in the environment and especially in (drinking) water is the potential evolution of antibiotic resistance, which poses a severe health risk to humans and animals [5,6,7,8,9]. From all classes of antibiotics, β-lactam antibiotics are prescribed most frequently, and particularly the aminopenicillin amoxicillin (AMX, Figure 1) is found among the top 25 of all prescribed drugs per year in each of the past 20 years [10,11,12,13].

As a consequence, AMX is traceable in many countries in hospital effluents, wastewater treatment plant influents as well as effluents, and eventually in surface waters which often serve as a source of drinking water [14,15,16,17,18,19,20,21]. In addition to the parent drug, further risk arises from the hydrolysis products of β-lactams, which can be formed after prolonged residence time in water. Even though antibiotic activity is lost upon hydrolysis, the formed hydrolysis products may still cause biological effects, e.g., allergenicity or ecotoxicity, that have not yet been investigated in detail [22,23]. Nevertheless, studies to identify those compounds and elucidate the mechanisms and kinetics of their formation have been carried out for the example of AMX [23,24,25,26,27,28].

In view of the numerous potential harmful metabolites that can be formed from β-lactams in the aquatic environment, effective screening for these compounds together with the unaltered parent antibiotic is required, especially since the Commission of the European Union has added AMX to a watch list of substances for Union-wide monitoring in the field of water policy [29]. Generally, mass spectrometry (MS)-based methods can be useful for this purpose as they enable highly accurate and sensitive determination of pharmaceutical compounds in water [30,31,32,33,34]. However, facing a large number of analytes, i.e., hydrolysis products of β-lactams, MS-based methods would be highly time-consuming and costly. Moreover, these instrumental methods require specially trained personnel as well as they are immobile and therefore not suitable for on-site testing. In contrast to this, immunochemical methods using specific antibodies allow for cost-effective on-site analysis with high throughput of samples and ease of experimentation. The standard technique in immunoassays can be considered the enzyme-linked immunosorbent assay (ELISA) which often serves as reference method for other assay formats. ELISAs for the determination of β-lactam antibiotics and particularly AMX have been developed previously but these studies focused on the determination of the unaltered drug and did not comprise studies on degradation products [35,36,37], even though the influence of β-lactam hydrolysis on antibody recognition has been reported before [38,39].

In this work, we present the first example of utilizing hydrolysis of β-lactams for the improved immunochemical determination of these compounds and their hydrolysis products, providing a method for the quick evaluation of drinking water safety and quality in terms of contamination with pharmaceuticals and therefore the risk of potential microbiological resistance development.

## 2. Results and Discussion

### 2.1. Hydrolysis of AMX

**Figure 2:**
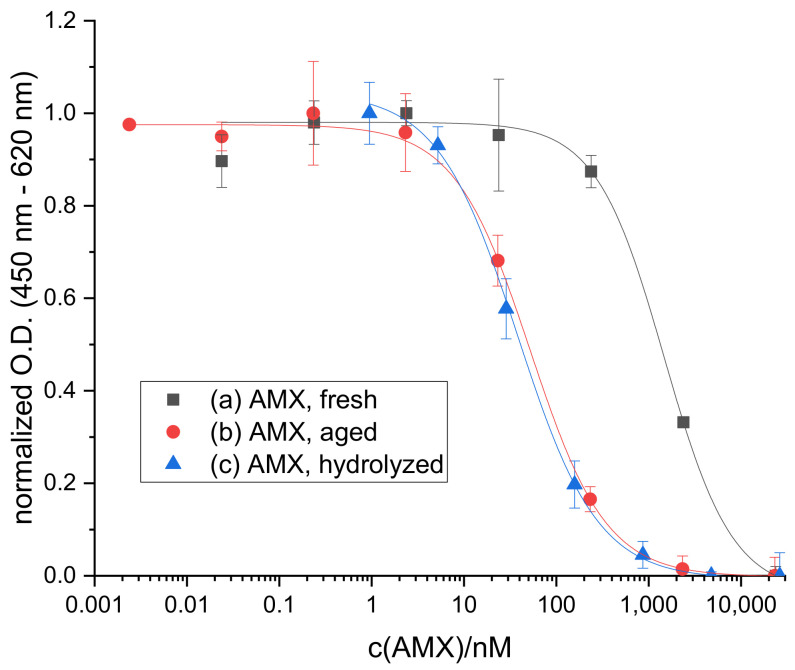
ELISA calibration curves obtained for standard solutions series of AMX after different treatments, (a) freshly prepared (reference), (b) aged for 2 months at 4 °C in a refrigerator, and (c) hydrolyzed during 24 h at 4 °C, also in a fridge. IC_50_ values: (a) 1470 ± 120 nM, (b) 52.2 ± 4.4 nM, and (c) 38.1 ± 3.6 nM.

While developing and optimizing an indirect competitive ELISA for the determination of AMX, we observed a significant shift in the calibration curves obtained for differently aged standard solutions (calibrators) of AMX in water. Interestingly, the curves for aged standard solutions were shifted to lower IC_50_ values, tantamount to a lower limit of detection. This shift reached its end point after two months of storage at 4 °C and could be ascribed to hydrolysis of the parent drug by hydrolyzing standard solutions of AMX in 0.1 M NaOH, which yielded a similar calibration curve in the ELISA (see Figure 2). Under these conditions (pH 12.5), it was found that hydrolysis had proceeded to a certain level because no further shift in the calibration curve was observable after reaction times of 18 h at 4 °C or 3 h at RT, respectively.

**Figure 3:**
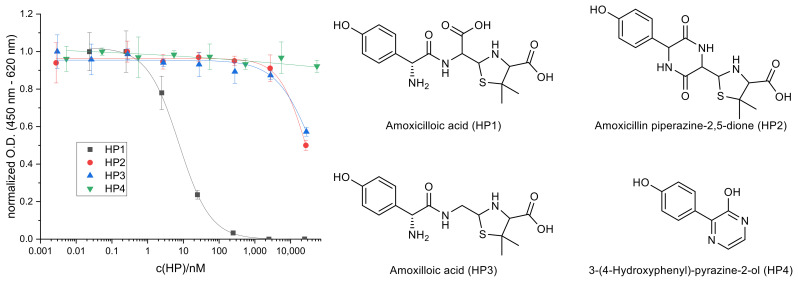
ELISA calibration curves of selected AMX hydrolysis products and their structures.

In order to discover the species that is recognized better by the antibody and therefore leads to the decreased IC_50_, several known hydrolysis products of AMX were synthesized or purchased as pure compounds and tested in the ELISA to compare their calibration curves. It was found that only the primary hydrolysis product amoxicilloic acid (HP1), which is formed by hydrolytic cleavage of the β-lactam ring, yields a complete inhibition curve in the examined concentration range (Figure 3). It is noteworthy that the IC_50_ of HP1 (7.48 ± 0.58 nM) is lower compared to hydrolyzed AMX, indicating that at least one additional species is formed in the hydrolyzed standards which shifts the calibration curve to an increased IC_50_ value again. Under the applied basic conditions, the formation of amoxicillin piperazine-2,5-dione (HP2, IC_50_ = 28,700 ± 1400 nM) by intramolecular ring closure of HP1 is feasible as well. The generation of the decarboxylation product amoxilloic acid (HP3) and 3-(4-hydroxyphenyl)-pyrazine-2-ol (HP4), which is considered a stable end product of AMX hydrolysis, are rather unlikely as their formation is favored under acidic conditions and elevated temperatures [23].

Given that the employed anti-AMX antibody shows an approximately 200-fold higher affinity towards HP1 than to the parent drug AMX, it appears probable that this antibody had been raised against HP1 rather than AMX. Depending on the immunogen synthesis strategy, i.e., the coupling of AMX to a carrier protein, the β-lactam ring could have been hydrolyzed prior to the immunization procedure. The producer of the antibody was contacted for details on the immunogen synthesis but classified this information as proprietary. However, as known from other immunogen syntheses, the coupling is often performed at alkaline pH allowing hydrolysis of the β-lactam ring to occur [36,38,40,41].

### 2.2. Cross-Reactivity of Related Penicillins

**Figure 4:**
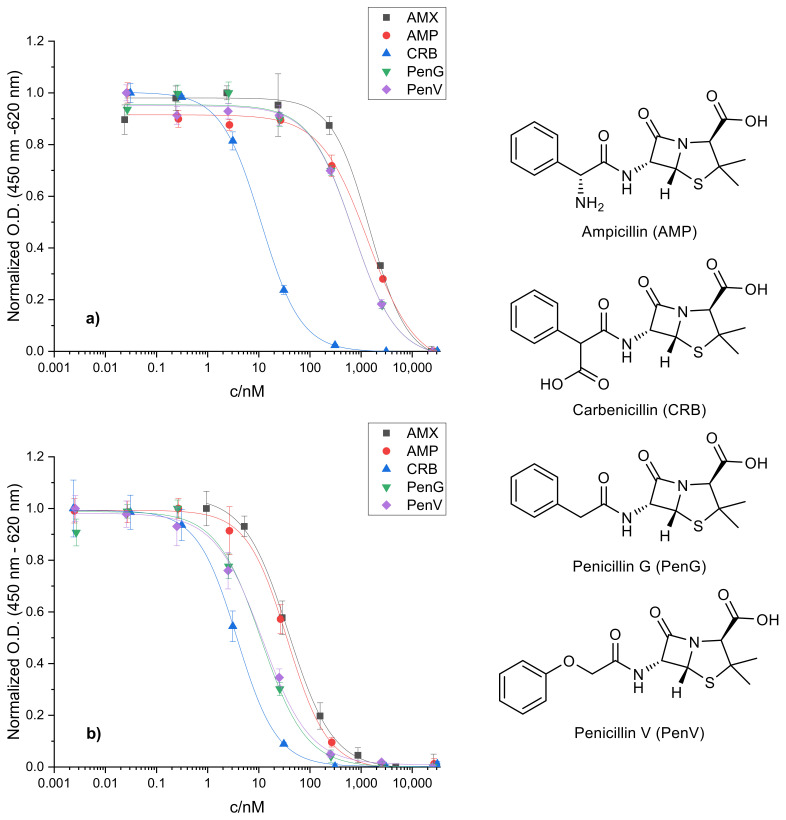
(**a**) ELISA calibration curves of structurally similar β-lactams before, and (**b**) after hydrolysis, and their structures (right). IC_50_ values (before; after hydrolysis): AMP (1350 ± 130 nM; 36.2 ± 3.7 nM), CRB (11.0 ± 0.4 nM; 3.8 ± 0.5 nM), PenG (700 ± 70 nM; 11.1 ± 1.7 nM), and PenV (702 ± 66 nM; 11.6 ± 2.5 nM).

A similar trend was observed for related penicillins that were tested for cross-reactivity. Regarding their structure, the following β-lactams were chosen: (1) ampicillin (AMP) which is lacking the aromatic hydroxyl group of AMX; (2) carbenicillin (CRB) with the α-amino group of AMP exchanged for a carboxyl group; (3) penicillin G (PenG) lacking any functional group in α-position; and (4) penicillin V (PenV) with an additional oxygen atom linking the phenyl ring to the α-carbon. ELISA calibration curves were recorded for each compound before and after alkaline hydrolysis of the respective standards and compared to fresh and hydrolyzed AMX standards (see Figure 4).

With freshly prepared, non-hydrolyzed standards, AMP yields a comparably high IC_50_ value in relation to AMX which is in accordance with their high structural similarity. PenG and PenV show very similar calibration curves, indicating that extension of the molecule by the oxygen atom has no effect on antibody recognition. However, both exhibit noticeably lower IC_50_ values than AMX and AMP. This suggests that the antibody binding is rather impaired than supported by the α-amino group which can be explained by electrostatic effects. Considering that the antibody was raised against HP1 of AMX (see above), which incorporates an additional, under the assay conditions negatively charged carboxyl group, the amino function of AMX and AMP would have the opposing effect, as it will become protonated and therefore positively charged. This is underlined by the exceptionally low IC_50_ obtained for CRB even prior to hydrolysis. CRB already bears an extra carboxyl group in the unhydrolyzed form supporting antibody binding. However, the exact position of the carboxyl group within the molecule appears to be secondary in this regard.

Interesting trends can be observed with hydrolyzed standards as well. Upon hydrolysis, the calibration curves of all regarded penicillins shift to lower IC_50_ values to a different extent. The IC_50_ of CRB is decreased just slightly, indicating that the second carboxyl group, which is formed by hydrolysis of the β-lactam ring, has a smaller supporting effect on antibody recognition. PenG and PenV behave similarly to each other after hydrolysis as well and their IC_50_ values match that of CRB before hydrolysis. Lastly, the calibration curve for hydrolyzed AMP resembles that of hydrolyzed AMX. As discussed for AMX (see above), AMP can not only form ampicilloic acid upon alkaline hydrolysis but also ampicillin diketopiperazine-2,5-dione due to its α-amino function. As shown for the analogous HP2 of AMX, the antibody’s affinity for this compound is clearly deteriorated. This could explain the higher IC_50_ of hydrolyzed AMP and AMX compared to hydrolyzed PenG and PenV since these cannot undergo intramolecular ring closure due to the lack of an α-amino group. Both will mainly form the respective penicilloic acid under the applied basic conditions. Therefore, the impact of hydrolysis on the antibody recognition is the largest for these two compounds leading to a reduction in the IC_50_ by 85% of the original value.

### 2.3. Sample Analysis

**Figure 5:**
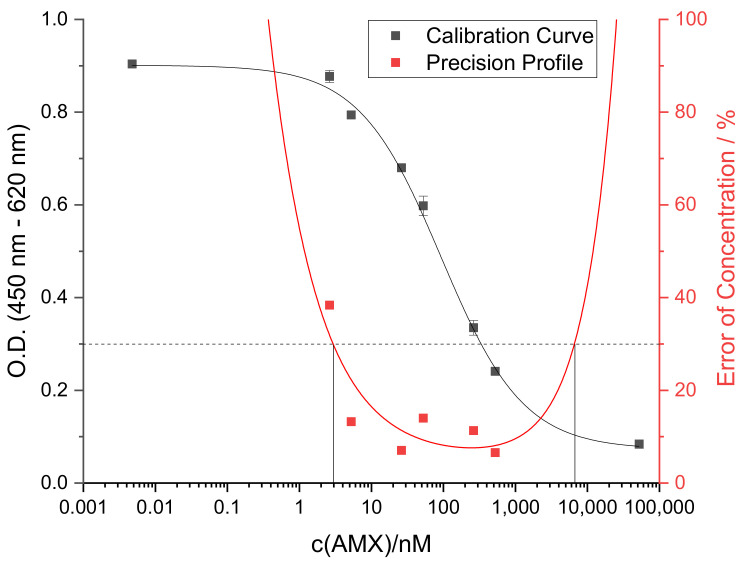
Calibration curve (black) and precision profile (red) for the quantification of AMX, indicating the measurement range of the assay to reach from AMX concentrations of 3 nM up to 7 µM.

From these preliminary studies, the following considerations have been made for the immunochemical determination of β-lactams in drinking water samples employing this antibody. Firstly, samples should be hydrolyzed prior to measurement in order to benefit from the enhanced affinity of the antibody for penicilloic acids. Secondly, the degree of hydrolysis in the sample can be estimated by performing a reference analysis in the assay without additional hydrolysis. For samples containing more than one β-lactam, this method can only provide qualitative or semi-quantitative results. However, for drinking water samples, tap water (TW) and mineral water (MW), spiked with known concentrations of the most abundant penicillin AMX, quantitative analyses were practicable. According to the precision profile of the assay (see Figure 5) which was determined as described by Ekins [42], the measurement range with an error of concentration below 30% reaches from 3 nM to 7 µM, so that various spiking concentrations within this range were chosen for validation of the assay.

**Figure 6:**
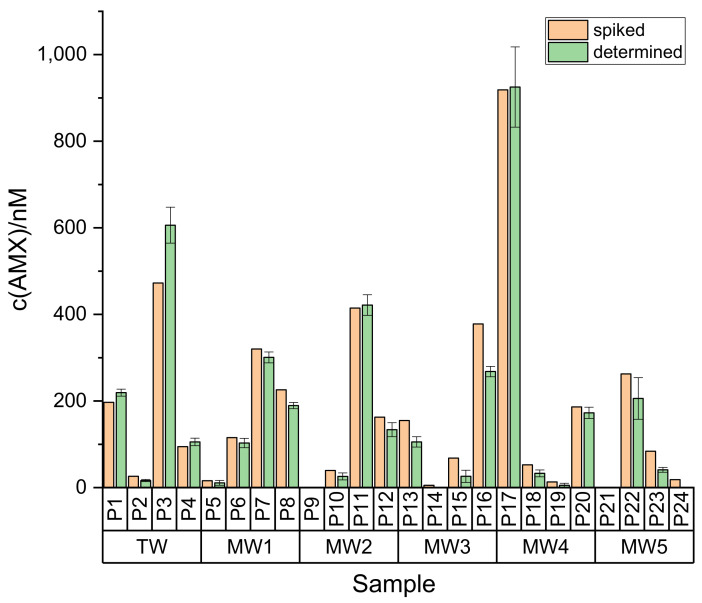
Comparison of spiked and determined concentrations of AMX in water samples. For details on sample parameters, see Supplementary Table S1.

As shown in Table 1 and Figure 6, the correlation between spiked and determined concentration of AMX is reasonable in this concentration range. However, at low AMX concentrations and for samples of high Ca^2+^ concentration (MW3 and MW5, see Supplementary Table S1), underdeterminations occurred, culminating in false negative results if both factors combine (P14 and P24). Blank samples (P9 and P21) were correctly identified (no false positive results).

In order to examine the degree of hydrolysis of AMX in these samples, a parallel analysis in the ELISA without pretreatment with 0.1 M NaOH was carried out and concentration values determined with and without hydrolysis were compared. As can be seen in Table 2, for most of the samples lower values were determined without hydrolysis, indicating that AMX is mainly unaltered in these samples. Because of the antibody’s lower affinity towards unhydrolyzed AMX (higher IC_50_), higher O.D. values are found which correlate with lower concentration values in the calibration curve. This was the case for all of the tested mineral waters after 20 h. Contrarily, for tap water samples (P1–P4) the analysis without hydrolysis led to higher concentration values than the assay featuring hydrolysis, implying that hydrolysis of AMX had already occurred in these samples. From this, it can be deduced that hydrolysis of AMX is much faster in tap water than in bottled mineral water under the test conditions. The reason for that may lie in general properties of tap water containing particles or metal ions, e.g., Cu^2+^, which can serve as catalysts for AMX hydrolysis [43]. Furthermore, the storage of the water samples might have an effect as tap water for sample preparation was collected and stored in a glass bottle whereas mineral water samples were taken directly from plastic bottles. Anyhow, the influence of the pH value of the water can be ruled out since the values were within a range from 6.6 to 8.0 and no general trend was observable. The same holds true for ionic constituents (see Supplementary Table S1).

## 3. Materials and Methods

### 3.1. Chemicals

Penicillin compounds amoxicillin trihydrate, ampicillin trihydrate, penicillin G potassium salt and penicillin V potassium salt were purchased as VETRANAL analytical standards from Sigma-Aldrich (Taufkirchen, Germany) or Riedel-de Haën (Seelze, Germany). Carbenicillin disodium salt was obtained from Serva (Heidelberg, Germany).

Hydrolysis products amoxicilloic acid and amoxicillin piperazine-2,5-dione were synthesized according to literature procedures [44,45]. Purity of the synthesis products was confirmed by elemental analysis, infrared (IR) spectroscopy and HPLC–MS analysis (see Supplementary Materials, Data S1). Penilloic acids of amoxicillin were purchased from LGC Standards (Luckenwalde, Germany) and 3-(4-hydroxyphenyl)pyrazin-2-ol (amoxicillin related compound F) was from Supelco (Bellefonte, PA, USA).

Buffer components were purchased from Sigma-Aldrich (Taufkirchen, Germany): sodium phosphate monobasic dihydrate, sodium phosphate dibasic dihydrate, sodium chloride, potassium phosphate monobasic, potassium phosphate dibasic, potassium sorbate, sodium citrate monobasic, tetrabutylammonium borohydride, ethylenediaminetetraacetic acid disodium salt dihydrate, *N*,*N*-dimethylacetamide anhydrous; Serva (Heidelberg, Germany): Tween 20, 3,3′,5,5′-tetramethylbenzidine; Merck (Darmstadt, Germany): tris(hydroxymethyl)-aminomethane, hydrochloric acid 32%; Fluka (Buchs, Switzerland: hydrogen peroxide solution 30%; and J.T. Baker (Phillipsburg, NJ, USA): sodium hydroxide, sulfuric acid 93–98%.

Ethanol (absolute, p. a., ACS, Ph. Eur., USP, min. 99.9%) was purchased from Th. Geyer (Renningen, Germany).

Immunoassay reagents amoxycillin-HSA (human serum albumin) conjugate (AMX-HSA) and amoxycillin-BSA (bovine serum albumin) conjugate (AMX-BSA) were from Squarix Biotechnology (Marl, Germany). Casein sodium salt from bovine milk was purchased from Sigma-Aldrich (Taufkirchen, Germany). Monoclonal mouse anti-AMX antibody A1463 (primary antibody, clone 1.BB.832, Lot: L11010709) was produced by US Biological (Salem, MA, USA). Polyclonal sheep anti-mouse IgG (H+L chain) antibody with HRP label (secondary antibody, R1256HRP) came from OriGene Technologies/Acris Antibodies (Herford, Germany).

### 3.2. Materials and Equipment

All ELISAs were performed in transparent 96-well f-bottom high-binding polystyrene microplates from Greiner Bio-One (Frickenhausen, Germany). Wells were filled by use of Eppendorf Research^®^ pro multichannel pipettes and dilutions of components were made with Research^®^ plus piston stroke pipettes from Eppendorf (Hamburg, Germany). For incubation, microplates were sealed with Parafilm^®^ from Bemis (Neenah, WI, USA) and shaken on a Titramax 101 orbital shaker from Heidolph Instruments (Schwabach, Germany). Washing steps were performed on a Microplate Washer 405 LS from BioTek Instruments (Winooski, VT, USA) and absorbance measurements on a SpectraMax Plus 384 microplate reader from Molecular Devices (San José, CA, USA).

Pure water was taken from a Merck Millipore Milli-Q Reference water purification system. Weighing was performed on a Sartorius (Göttingen, Germany) Research R180D-*D1 analytical balance. Measurements of pH values were carried out with a SevenEasy pH meter S20 from Mettler Toledo (Columbus, OH, USA).

### 3.3. Buffers

All buffers were prepared in Milli-Q water and stored in amber glass bottles at room temperature (RT, 22 ± 1 °C) unless stated otherwise. The pH values were adjusted with 6 M hydrochloric acid or 5 M sodium hydroxide solution.

- Phosphate-buffered saline (PBS), pH 7.6: 10 mM sodium phosphate monobasic dihydrate, 70 mM sodium phosphate dibasic dihydrate, 145 mM sodium chloride.
- Washing buffer 60×, pH 7.6: 45 mM potassium phosphate monobasic, 375 mM potassium phosphate dibasic, 1.5 mM potassium sorbate, 3% Tween 20.
- Tris-buffered saline (Tris), pH 8.5: 10 mM tris(hydroxymethyl)aminomethane, 150 mM sodium chloride.
- Sample buffer, pH 7.6 or pH 8.5: 125 mM tris(hydroxymethyl)aminomethane, 187.5 mM sodium chloride, 13.375 mM ethylenediaminetetraacetic acid disodium salt dihydrate.
- Citrate buffer, pH 4.0, storage at 4 °C: 220 mM sodium citrate monobasic.
- TMB stock solution in *N*,*N*-dimethylacetamide, storage at 4 °C under argon: 8 mM tetrabutylammonium borohydride, 40 mM 3,3′,5,5′-tetramethylbenzidine (TMB).

### 3.4. Standards and Samples

For the preparation of standards (calibrators), stock solutions of each compound with a mass concentration of approximately 1 g/L were prepared gravimetrically by weighing the respective compound and amount of solvent. Solvents for these stock solutions were ethanol for amoxicillin piperazine-2,5-dione and 3-(4-hydroxyphenyl)pyrazin-2-ol, or Milli-Q water for all other compounds. Standard solutions of each compound were prepared volumetrically by serial dilution of the respective stock solution in Milli-Q water. All standards and stock solutions were stored at 4 °C in the amber glass vials.

Spiked drinking water samples of AMX were prepared by further diluting a prediluted stock solution of amoxicillin trihydrate in Milli-Q water in the respective water sample (tap water or bottled mineral water). Tap water was taken from a water cooler at our institute and collected in a glass bottle. Non-sparkling mineral waters were purchased in plastic bottles and used directly for sample preparation.

For hydrolysis, standards and samples were diluted in 0.1 M sodium hydroxide solution (1:1) the day before analysis in the assay, i.e., the day of coating (see below), and stored at 4 °C until the next day. Samples for reference analysis without hydrolysis were diluted in Milli-Q water (1:1) prior to analysis.

### 3.5. Immunoassay Procedure

Each cavity of a 96-well microplate was coated with AMX-HSA (or alternatively AMX-BSA) in PBS (47.619 ng/L, 200 µL/well) and incubated for 18 h at RT with shaking at 750 rpm. Afterwards the plate was washed three times with washing buffer (1:60 dilution of washing buffer 60x in Milli-Q water) and the cavities were blocked with 0.1% (*w*/*v*) casein in PBS (200 µL/well) for 1 h at 750 rpm and RT. After repeated washing, standards/samples (100 µL/well) and primary antibody (dilution 1:20,000 in Tris for preliminary and cross-reactivity studies, in sample buffer pH 7.6 for sample analysis or in sample buffer pH 8.5 for reference analysis for degree of hydrolysis; 100 µL/well) were added and incubated for 1 h at 750 rpm and RT. Following another washing step, the plate was incubated with secondary antibody (95.238 ng/L, 200 µL/well) for 1 h at 750 rpm at RT. After final washing, cavities were filled with freshly prepared substrate solution (22 mL citrate buffer, 8.5 µL hydrogen peroxide solution, 550 µL TMB stock solution; 200 µL/well) and shaken for 10 min (preliminary and cross-reactivity studies) or 20 min (sample analysis, degree of hydrolysis) at 750 rpm for blue color development. The reaction was stopped by adding 1 M sulfuric acid (100 µL/well) and shaking for 1 min which resulted in color change of the solutions from blue to yellow. Optical density was read at RT at a wavelength of 450 nm with reference at 620 nm. Data points were plotted in Origin^®^ 2019 (OriginLab, Northampton, MA, USA), calibration curves were obtained by fitting a four-parameter logistic function to the measured data points and sample concentrations were determined by correlating concentration values of hydrolyzed standards with O.D. values of hydrolyzed and non-hydrolyzed samples. Each standard (8 per plate) and sample (24 per plate) was analyzed in triplicate with random distribution across the plate to reduce edge effects.

## 4. Conclusions

In summary, it can be said that the immunochemical determination of β-lactam antibiotics in water is not as straightforward as previous works in this field suggest. Several considerations have to be made in order to obtain the best analytical results. The choice of the antibody is crucial as we have shown for a commercial anti-AMX antibody that exhibits a higher affinity not only towards a hydrolysis product of AMX but also to other penicillins and their hydrolysis products. This might be true for other anti-penicillin antibodies on the market as well. The production of high-affinity, specific penicillin antibodies should comprise careful consideration of the immunogen synthesis in order to prevent hydrolysis of the β-lactam during this step. Furthermore, the use of non-hydrolyzable structural mimics of these compounds for immunization might be an interesting strategy as well. Also, the production of antibodies via recombinant expression appears to be promising since recombinant techniques enable controlled and reliable antibody engineering [46].

Nevertheless, we were able to evolve a strategy for water analysis with the available antibody in terms of AMX quantification and assessment of the hydrolysis degree in the samples. Based on our findings, this strategy could also be transferable to other water-based matrices, e.g., milk and blood, and expandable to enzymatic cleavage of penicillins by β-lactamases during sample preparation [38].

Our future efforts will focus on transferring this system to other platforms, such as magnetic bead-based assays and eventually accomplishing the transition from a plate-based assay to an integrated immunosensor suitable for online sensing applications in drinking water supply systems. Additionally, the production of more sensitive and specific antibodies for AMX by recombinant techniques is considered for the future.

## Figures and Tables

**Table 1:** Results of AMX determination in spiked water samples by ELISA.

Sample	c(AMX)/nM		Recovery
	Spiked	Determined	
P1	197	219 ± 8	111%
P2	26.2	16.3 ± 2.6	62%
P3	472	606 ± 41	128%
P4	94.5	105 ± 9	112%
P5	15.8	11.0 ± 5.6	70%
P6	115	103 ± 11	89%
P7	320	301 ± 13	94%
P8	226	189 ± 7	84%
P9	0	0	100%
P10	39.4	26.0 ± 8.1	66%
P11	415	422 ± 24	102%
P12	163	134 ± 16	82%
P13	155	106 ± 12	68%
P14	5.25	0	0%
P15	68.2	26.1 ± 14.2	38%
P16	378	268 ± 12	71%
P17	919	925 ± 93	101%
P18	52.5	32.9 ± 8.0	63%
P19	13.1	5.14 ± 4.8	39%
P20	186	173 ± 13	93%
P21	0	0	100%
P22	262	206 ± 48	78%
P23	84.0	41.0 ± 5.6	49%
P24	18.4	0	0%

**Table 2:** Comparison of AMX concentrations determined by ELISA with and without hydrolysis allowing for assessment of AMX hydrolysis degree in the water samples (“+”: hydrolyzed; “-”: unhydrolyzed; “0”: blank/false negative).

Sample	c(AMX) Determined/nM		+/-
	With Hydrolysis	Without Hydrolysis	
P1	219	333	+
P2	16.3	28.3	+
P3	606	1430	+
P4	105	169	+
P5	11.0	0	-
P6	103	2.81	-
P7	301	12.6	-
P8	189	6.33	-
P9	0	0	0
P10	26.0	0	-
P11	422	10.2	-
P12	134	2.71	-
P13	106	0	-
P14	0	0	0
P15	26.1	0	-
P16	268	13.3	-
P17	925	31.3	-
P18	32.9	0	-
P19	5.14	0	-
P20	173	5.56	-
P21	0	0	0
P22	206	4.38	-
P23	41.0	0	-
P24	0	2.85	0

## Data Availability

The data presented in this study are available on request from the corresponding author. The data are not publicly available due to constraints stipulated in the cooperation agreement between project partners.

## Supplementary Materials

The following are available online at https://www.mdpi.com/2079-6382/10/3/298/s1, Data S1: Synthesis and characterization of amoxicillin hydrolysis products; Table S1: Water sample parameters.
