# Supplementary material for: Pitfalls in the Immunochemical Determination of β-Lactam Antibiotics in Water
Source: Antibiotics (Basel). 2021 Mar 12;10(3):298. doi: 10.3390/antibiotics10030298 (PMC8001000; doi:10.3390/antibiotics10030298)
Supplement: Supplementary file 1 [file antibiotics-10-00298-s001.pdf]

antibiotics

Supplementary

Pitfalls in the Immunochemical Determination of β-Lactam Antibiotics in Water

Alexander Ecke, Rudolf J. Schneider and Supplementary Information

Data S1. Synthesis and characterization of amoxicillin hydrolysis products.

Amoxicilloic acid (HP1) was synthesized as described by MUNRO et al. and recovered as the respective monosodium salt monohydrate [1].

HPLC (H_2O, 10 µL, 350 µL/min, 30 °C, 98% MeCN, 2% 10 mM $NH_4(HCOO)$ in H_2O): t_R = 7.439, 8.361 min (two enantiomers).

MS (ESI, 450 °C, 1200 V, pos.): m/z = 384.30 ($[HP1+H]^+$, calc. 384.12), 367.29 ($[HP1\text{-}NH_2]^+$, calc. 367.10), 340.40 ($[HP1\text{-}CO_2+H]^+$, calc. 340.13), 323.30 ($[HP1\text{-}CO_2\text{-}NH_2]^+$, calc. 323.11), 189.22, 160.12, 114.20, 100.22.

Elemental analysis ($C_{16}H_{22}N_3O_7SNa$, M = 423.42 g/mol):
calc.: C 45.39, H 5.24, N 9.92, S 7.57%
found: C 45.00, H 5.29, N 9.75, S 7.12 %.

IR (CaF_2): $\tilde{\nu}$ = 3497 (m), 3360 (s), 3273 (s), 3072 (m), 1687 (vs), 1605 (vs), 1521 (s), 1493 (m), 1454 (m), 1388 (vs), 1286 (w), 1264 (m), 1248 (w), 1229 (w), 1212 (w), 1185 (w), 836 (m), 762 cm^{-1} (s).

Amoxicillin piperazine-2,5-dione (HP2) was synthesized in analogy to the related ampicillin piperazine-2,5-dione as described by BUNDGAARD et al. [2].

HPLC (EtOH, 10 µL, 350 µL/min, 30 °C, 97.902% MeCN, 1.998% H_2O, 0.1% HCOOH: t_R = 11.399 min.

MS (ESI, 450 °C, 1200 V, pos.): m/z = 366.21 ($[HP2+H]^+$, calc. 366.11), 207.20, 160.16, 114.14; 100.17.

Elemental analysis ($C_{16}H_{19}N_3O_5S$, M = 365.40 g/mol):
calc.: C 52.59, H 5.24, N 11.50, S 8.77 %
found: C 52.35, H 5.27, N 11.25, S 8.74 %.

IR (CaF_2): $\tilde{\nu}$ = 3425 (s), 3184 (s), 2859 (s), 1736 (s), 1667 (vs), 1614 (m), 1599 (m), 1559 (w), 1519 (m), 1451 (m), 1389 (w), 1372 (m), 1342 (w), 1302 (w), 1284 (m), 1272 (m), 1256 (m), 1208 (m), 1193 (m), 1172 (m), 1157 (w), 1135 (m), 1107 (w), 1091 (w), 828 (s), 746 cm^{-1} (vs).

LC-MS analyses were performed on an *API 2000* Linear Ion Trap Quadrupole LC-MS/MS mass spectrometer from AB Sciex Instruments with an Agilent *1200* liquid chromatograph equipped with a *Kinetex*

XB-C18 column (2.6 µm, 150 mm x 3 mm) with UHPLC C18, 3 mm column guard from Phenomenex.

Elemental analysis was executed on a *Euro EA 3000* CHNS elemental analyzer from HEKAtech.

IR spectra were recorded on a Bruker *Equinox 55* with IR-microscope *Scope II*.

Table S1. Water sample parameters. Data for ionic constituents were provided by the manufacturers, and pH values were determined with a pH electrode (n. d = not determined).

Sample	pH	Concentration of ionic constituents/(mg/L)								
		Na^+	K^+	Mg^{2+}	Ca^{2+}	Cl^-	SO_4^{2-}	HCO_3^-	F^-	H_2SiO_3
**TW**	7.95	38.0	4.9	10.7	108	55.0	117	250	n. d.	n. d.
**MW1**	7.83	4.4	0.7	5.0	57.2	6.0	n. d.	186	0.12	n. d.
**MW2**	6.64	6.2	1.7	3.3	17.0	5.1	56.9	13.2	n. d.	n. d.
**MW3**	7.87	10.8	1.6	62.1	373	n. d.	900	266	n. d.	13.1
**MW4**	7.07	6.8	1.5	3.1	16.2	5.2	55.0	8.0	n. d.	n. d.
**MW5**	6.65	71.5	9.2	92.2	235	241	499	333	n. d.	n. d.

References

1. Munro, A.C.; Chainey, M.G.; Woroniecki, S.R. Preparation and immunological cross-reactions of penicilloic and penilloic acids. *J. Pharm. Sci.* **1978**, *67*, 1197-1204, doi:10.1002/jps.2600670903.
2. Bundgaard, H.; Larsen, C. Piperazinedione formation from reaction of ampicillin with carbohydrates and alcohols in aqueous solution. *Int. J. Pharm.* **1979**, *3*, 1-11, doi:10.1016/0378-5173(79)90044-9.
